# Supplementary material for: Wild Nutria (Myocastor coypus) Is a Potential Reservoir of Carbapenem-Resistant and Zoonotic Aeromonas spp. in Korea
Source: Microorganisms. 2019 Jul 30;7(8):224. doi: 10.3390/microorganisms7080224 (PMC6723217; doi:10.3390/microorganisms7080224)
Supplement: Supplementary file 1 [file microorganisms-07-00224-s001.pdf]

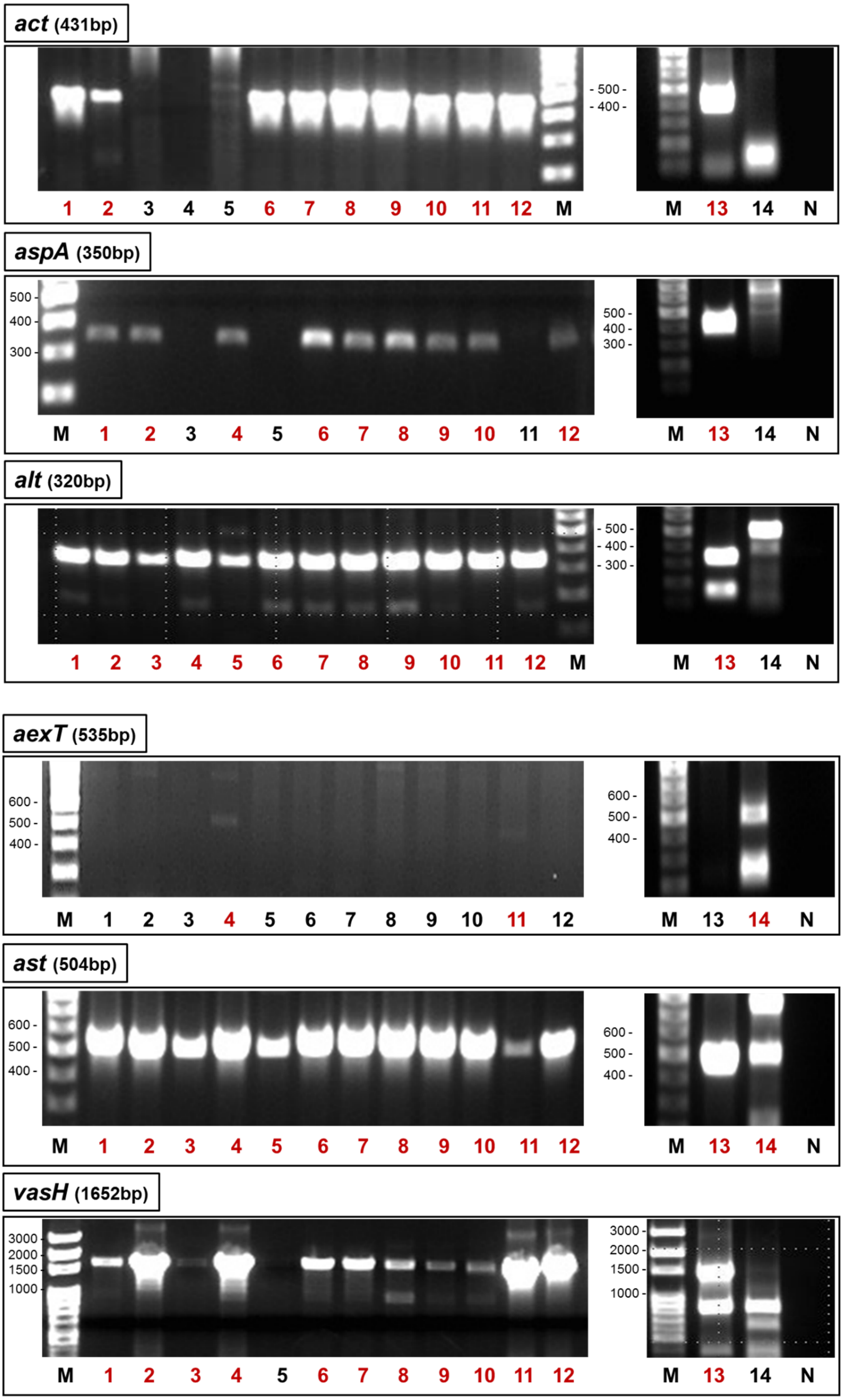
act (431bp)
- 500 -
- 400 -
1 2 3 4 5 6 7 8 9 10 11 12 M
M 13 14 N
aspA (350bp)
500 -
400 -
300 -
M 1 2 3 4 5 6 7 8 9 10 11 12
M 13 14 N
alt (320bp)
- 500 -
- 400 -
- 300 -
1 2 3 4 5 6 7 8 9 10 11 12 M
M 13 14 N
aexT (535bp)
600 -
500 -
400 -
M 1 2 3 4 5 6 7 8 9 10 11 12
M 13 14 N
ast (504bp)
600 -
500 -
400 -
M 1 2 3 4 5 6 7 8 9 10 11 12
M 13 14 N
vasH (1652bp)
3000 -
2000 -
1500 -
1000 -
M 1 2 3 4 5 6 7 8 9 10 11 12
M 13 14 N

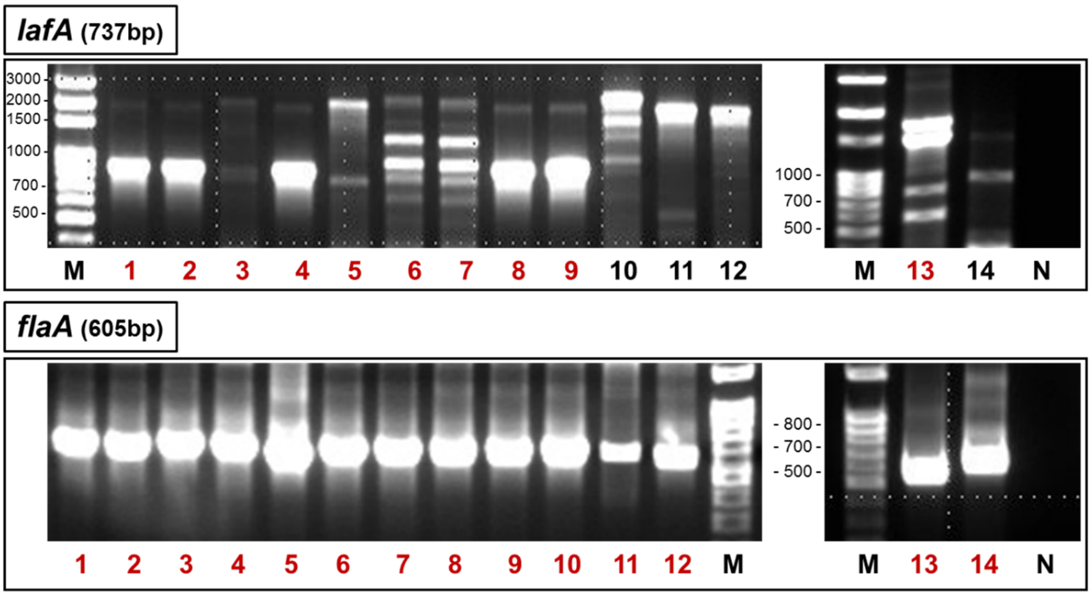

Supplementary Figure S1. Gel electropherogram of virulence-related gene amplicons from the 14 *Aeromonas* isolates used in this study. Virulence genes showing positive amplicons are only shown and red numbers indicate positive amplicons, respectively. *Aeromonas* strains were arranged in the following order: 1, *A. hydrophila* KN-Mc-1R1; 2, *A. hydrophila* KN-Mc-1R2; 3, *A. cavieae* KN-Mc-1R3; 4, *A. hydrophila* KN-Mc-2R1; 5, *A. cavieae* KN-Mc-3R1; 6, *A. hydrophila* KN-Mc-4N1; 7, *A. hydrophila* KN-Mc-4N3; 8, *A. hydrophila* KN-Mc-5R1; 9, *A. hydrophila* KN-Mc-5R2; 10, *A. hydrophila* KN-Mc-6U21; 11, *A. dhakensis* KN-Mc-6U2; 12, *A. hydrophila* KN-Mc-6U22; 13, *A. hydrophila* KN-Mc-10N1; 14, *A. rivipollensis* KN-Mc-11N1; N, negative control; M, SiZer™-100 plus DNA marker (Intron Biotech, Seongnam, Republic of Korea).

Supplementary Table S1. List of PCR primers used in this study.

Gene	Primer sequence (5′ to 3′)	Ref
Bacterial identification (Product size (bp))		
16S rRNA (1466)	F(27F): AGAGTTTGATCMTGGCTCAG<br>R(1492R): TACGGYTACCTTGTTACGACTT	Universal primer
gyrB (≈1100)	F(gyrB3F): TCCGGCGGTCTGCACGGCGT<br>R(gyrB14R): TTGTCCGGGTTGTACTCGTC	[1]
rpoB (560)	F(Pasrpob-L): GCAGTGAAAGARTTCTTTGGTTC<br>R(Rpob-R): GTTGCATGTTNGNACCCAT	[2]
Virulence-associated genes (Product size (bp))		
Act (431)	F: CCTATGGCCTGAGCGAGAAG<br>R: CCAGTTCCAGTCCCACCACT	[3]
aexT (535)	F: GGCGCTTGGGCTCTACAC<br>R: GAGCCCGCGCATCTTCAG	[4]
Alt (320)	F: AAAGCGTCTGACAGCGAAGT<br>R: AGCGCATAGGCGTTCTCTT	[5]
ascV (710)	F: ATGGACGGCGCCATGAAGTT<br>R: TATTCGCCTTCACCCATCCC	[6]
aspA (350)	F: CACCGAAGTATTGGGTCAGG<br>R: GGCTCATGCGTAACTCTGGT	[3]
Ast (504)	F: ATCGTCAGCGACAGCTTCTT<br>R: CTCATCCCTTGGCTTGTTGT	[5]
BfpA (251)	F: CCGCAGGTGTGATGTTTTAC<br>R: TGCGGTGTTATTGTTTGCT	[7]
BfpG (233)	F: ATGCCAAAGCTGACTGGTCT<br>R: GACATGATTCCCGTTATAAA	[7]
flaA (608)	F: TCCAACCGTYTGACCTC<br>R: GMYTGGTTGCGRATGGT	[8]
lafA (737)	F: CCAACTTYGCYTCYMTGACC<br>R: TCTTGGTCATRTTGGTGCTY	[5]
stx-1 (180)	F: ATAAATTGCCATTCGTTGACTAC<br>R: AGAACGCCCACTGAGATCATC	[9]

	stx-2 (255)	F: GGCACTGCTTGAAACTGCTCC<br>R: TCGCCAGTTATCTGACATTCTG	[9]
	vasH (1,652)	F: GCTCTAGACCGGTGAACCCATCAAGCGCGTCCACT<br>R: TCCCCCCGGGCTGGTGGCCAGCAGCAGAGGCAATA	[10]
Antimicrobial-resistance genes (Product size (bp))			
T*	*tetA* (211)	F(*tet*AF): GCTACATCCTGCTTGCCTTC<br>R(*tet*AR): GCATAGATCGCCGTGAAGAG	[11]
	tetB (391)	F(ClassB *tet*AF): TCATTGCCGATACCACCTCAG<br>R(ClassB *tet*AR): CCAACCATCATGCTATTCCATCC	
	tetC (897)	F(ClassC *tet*AF): CTGCTCGCTTCGCTACTTG<br>R(ClassC *tet*AR): GCCTACAATCCATGCCAACC	
	tetD (844)	F(ClassD *tet*AF): TGTGCTGTGGATGTTGTATCTC<br>R(ClassD *tet*AR): CAGTGCCGTGCCAATCAG	
	tetE (744)	F(ClassE *tet*AF): ATGAACCGCACTGTGATGATG<br>R(ClassE *tet*AR): ACCGACCATTACGCCATCC	
Q*	*gyrA* (663)	F(ASGYRA1): CCATGAGCGTGATCGTAGGA<br>R(ASGYRA2): CTTTGGCACGCACATAGACG	[12]
	parC (418)	F(ASPARC3): CAGCGGCGCATCATCTAC<br>R(ASPARC4): GGATATCGGTGGCCATGC	
	qnrA1 to *qnrA6* (580)	F(qnrAm-F): AGAGGATTTCTCACGCCAGG<br>R(qnrAm-R): TGCCAGGCACAGATCTTGAC	[13]
	qnrB1 to *qnrB6* (264)	F(qnrBm-F): GGMATHGAAATTCGCCACTG<br>R(qnrBm-R): TTTGCYGYYCGCCAGTCGAA	
	qnrS1 to *qnrS2* (428)	F(qnrSm-F): GCAAGTTCATTGAACAGGGT<br>R(qnrSm-R): TCTAAACCGTCGAGTTCGGCG	
I*	Class 1 integron (0.7~3.0 k)	F(5'-CS): GGCATCCAAGCAGCAAG<br>R(3'-CS): AAGCAGACTTGACCTGA	[14]
B*	CTX-M-1 group (688)	F(CTXGp1-F): TTAGGAARTGTGCCGCTGYA<br>R(CTXGp1-R): CGATATCGTTGGTGGTRCCAT	[15]
	CTX-M-2 group (404)	F(CTXGp2-F): CGTTAACGGCACGATGAC<br>R(CTXGp2-R): CGATATCGTTGGTGGTRCCAT	
	CTX-M-9 group (561)	F(CTXGp9-F): TCAAGCCTGCCGATCTGGT<br>R(CTXGp9-R): TGATTCTCGCCGCTGAAG	
	TEM (800)	F(TEM-F): CATTTCCGTGTCGCCCTTATTC<br>R(TEM-R): CGTTCATCCATAGTTGCCTGAC	
	SHV (713)	F(SHV-F): AGCCGCTTGAGCAAATTAAAC<br>R(SHV-R): ATCCCGCAGATAAATCACCAC	
	OXA-A variants (564)	F(OXAA-F): GGCACCAGATTCAACTTTCAAG<br>R(OXAA-R): GACCCCAAGTTTCCTGTAAGTG	
	MOX-1, MOX-2, CMY-1, CMY-8 to -11 (520)	F(MOXMF): GCTGCTCAAGGAGCACAGGAT<br>R(MOXMR): CACATTGACATAGGTGTGGTGC	[16]
	LAT-1 to -4, CMY-2 to -7, BIL-1 (462)	F(CITMF): TGGCCAGAACTGACAGGCAAA<br>R(CITMR): TTTCTCCTGAACGTGGCTGGC	
	DHA-1 to -2 (405)	F(DHAMF): AACTTTCACAGGTGTGCTGGGT<br>R(DHAMR): CCGTACGCATACTGGCTTTGC	
	ACC (346)	F(ACCMF): AACAGCCTCAGCAGCCGGTTA<br>R(ACCMR): TTCGCCGCAATCATCCCTAGC	
	MIR-1, ACT-1 (302)	F(EBCMF): TCGGTAAAGCCGATGTTGCGG<br>R(EBCMR): CTTCCACTGCGGCTGCCAGTT	
	FOX-1 to -5b (190)	F(FOXMF): AACATGGGGTATCAGGGAGATG<br>R(FOXMR): CAAAGCGCGTAACCGGATTGG	
C*	*cphA* (≈720)	F(ANY-SSD/F):GCTTAGAGCTCCTAAGGAGCAAGATGAAAGGTTGG<br>R(ANI-SSD/R):GCATAGGTACCTTATGACTGGGGTGCGGCCTTG	[17]

* T, tetracycline; Q, quinolones; I, integrons; B, β-lactams; C, carbapenems.

Supplementary Table S2. Biochemical characterization of *Aeromonas* spp. used in this study.

	Strains													
	KN-Mc-1R1	KN-Mc-1R2	KN-Mc-1R3	KN-Mc-2R1	KN-Mc-3R1	KN-Mc-4N1	KN-Mc-4N3	KN-Mc-5R1	KN-Mc-5R2	KN-Mc-6U2	KN-Mc-6U21	KN-Mc-6U22	KN-Mc-10N1	KN-Mc-11N1
ONPG	+	+	+	+	+	+	+	-	-	+	+	+	+	+
ADH	+	+	+	+	+	+	+	+	+	+	+	+	+	+
LDC	+	+	-	+	-	+	+	+	+	+	+	+	+	-
ODC	-	-	-	-	-	-	-	-	-	-	-	-	-	-
CIT	-	-	-	-	-	-	-	-	-	-	-	-	-	-
H_2S	-	-	-	-	-	-	-	-	-	-	-	-	-	-
URE	-	-	-	-	-	-	-	-	-	-	-	-	-	-
TDA	+	+	+	+	+	+	+	+	+	+	+	+	+	+
IND	+	+	+	+	+	+	+	+	+	+	+	+	+	+
VP	+	+	-	+	-	+	+	+	+	+	+	+	+	-
GEL	+	+	+	+	+	+	+	+	+	+	+	+	+	-
GLU	+	+	+	+	+	+	+	+	+	+	+	+	+	-
MAN	+	+	+	+	+	+	+	+	+	+	+	+	+	+
INO	-	-	-	-	-	-	-	-	-	-	-	-	-	-
SOR	-	-	-	-	-	-	-	-	-	-	-	-	-	-
RHA	-	-	-	-	-	-	-	-	-	+	-	-	-	-
SAC	+	+	+	+	+	+	+	+	+	+	+	-	+	+
MEL	-	-	-	-	-	-	-	-	-	-	-	-	-	-
AMY	-	-	+	-	+	+	+	-	-	-	-	-	-	-
ARA	+	+	+	+	+	+	+	+	+	+	-	+	+	+

ONPG; β-galactosidase, ADH; arginine dihydrolase, LDC; lysine decarboxylase, ODC; ornithine decarboxylase, CIT; citrate utilization, H_2S; H_2S production, URE; urease, TDA; tryptophane deaminase, IND; indole production, VP; Voges–Proskauer, GEL; gelatinase, GLU; glucose, MAN; mannitol, INO; inositol, SOR; sorbitol, RHA; rhamnose, SAC; saccharose, MEL; melibiose, AMY; amygdalin, ARA; arabinose.

Reference

1. Yanez, M.A.; Catalán, V.; Apraiz, D.; Figueras, M.J.; Martinez-Murcia, A.J. Phylogenetic analysis of members of the genus *Aeromonas* based on *gyrB* gene sequences. *Int J Syst Evol Microbiol*. **2003**, *53*, 875–883. https://doi.org/10.1099/ijs.0.02443-0 PMID: 12807216.
2. Korczak, B.; Christensen, H.; Emler, S.; Frey, J.; Kuhnert, P. Phylogeny of the family *Pasteurellaceae* based on *rpoB* sequences. *Int J Syst Evol Microbiol*. **2004**, *54*, 1393–1399. https://doi.org/10.1099/ijs.0.03043-0 PMID: 15280320.
3. Soler, L.; Figueras, M.J.; Chacón, M.R.; Vila, J.; Marco, F.; Martinez-Murcia, A.J.; Guarro, J. Potential virulence and antimicrobial susceptibility of *Aeromonas popoffii* recovered from freshwater and seawater. *FEMS Immunol Med Microbiol*. **2002**, *32*, 243-247. https://doi.org/10.1111/j.1574-695X.2002.tb00560.x PMID: 11934570.
4. Braun, M.; Stuber, K.; Schlatter, Y.; Wahli, T.; Kuhnert, P.; Frey, J. Characterization of an ADP-ribosyltransferase toxin (AexT) from *Aeromonas salmonicida* subsp. *salmonicida. J Bacteriol*. **2002**, *184*, 1851-1858. https://doi.org/10.1128/jb.184.7.1851-1858.2002 PMID: 11889090.
5. Aguilera-Arreola, M.G.; Hernández-Rodríguez, C.; Zúñiga, G.; Figueras, M.J.; Castro-Escarpulli G. *Aeromonas hydrophila* clinical and environmental ecotypes as revealed by genetic diversity and virulence genes. *FEMS Microbiol Lett*. **2005**, *242*, 231–240. https://doi.org/10.1016/j.femsle.2004.11.011 PMID: 15621443.
6. Chacón, M.R.; Soler, L.; Groisman, E.A.; Guarro, J.; Figueras, M.J. Type III secretion system genes in clinical *Aeromonas* isolates. *J Clin Microbiol*. **2004**, *42*, 1285-1287. https://doi.org/10.1128/jcm.42.3.1285-1287.2004 PMID: 15004096.
7. Sechi, L.A.; Deriu, A.; Falchi, M.P.; Fadda, G.; Zanetti, S. Distribution of virulence genes in *Aeromonas* spp. isolated from Sardinian waters and from patients with diarrhoea. *J Appl Microbiol*. **2002**, *92*, 221-227. https://doi.org/10.1046/j.1365-2672.2002.01522.x PMID: 11849349.
8. Sen, K.; Rodgers, M. Distribution of six virulence factors in *Aeromonas* species isolated from US drinking water utilities: a PCR identification. *J Appl Microbiol*. **2004**, *97*, 1077-1086. https://doi.org/10.1111/j.1365-2672.2004.02398.x
9. Paton, A.W.; Paton, J.C. Detection and characterization of Shiga toxigenic *Escherichia coli* by using multiplex PCR assays for *stx*$_1$, *stx*$_2$, *eaeA*, enterohemorrhagic *E. coli hlyA*, *rfb*$_{O111}$, and *rfb*$_{O157}$. *J Clin Microbiol*. **1998**, *36*, 598-602. PMID: 9466788
10. Suarez, G.; Sierra, J.C.; Sha, J.; Wang, S.; Erova, T.E.; Fadl, A.A.; Foltz, S.M.; Horneman, A.J.; Chopra, A.K. Molecular characterization of a functional type VI secretion system from a clinical isolate of *Aeromonas hydrophila*. *Microb Pathog*. **2008**, *44*, 344-361. https://doi.org/10.1016/j.micpath.2007.10.005 PMID: 18037263.
11. Nawaz, M.; Sung, K.; Khan, S.A.; Khan, A.A.; Steele, R. Biochemical and molecular characterization of tetracycline-resistant *Aeromonas veronii* isolates from catfish. *Appl Environ Microbiol*. **2006**, *72*, 6461-6466. https://doi.org/10.1128/AEM.00271-06 PMID: 17021193.
12. Giraud, E.; Blanc, G.; Bouju-Albert, A.; Weill, F.X.; Donnay-Moreno, C. Mechanisms of quinolone resistance and clonal relationship among *Aeromonas salmonicida* strains isolated from reared fish with furunculosis. *J Med Microbiol*. **2004**, *53*, 895-901. https://doi.org/10.1099/jmm.0.45579-0 PMID: 15314197.
13. Cattoir, V.; Poirel, L.; Rotimi, V.; Soussy, C.J.; Nordmann, P. Multiplex PCR for detection of plasmid-mediated quinolone resistance *qnr* genes in ESBL-producing enterobacterial isolates. *J Antimicrob Chemother*. **2007**, *60*, 394-397. https://doi.org/10.1093/jac/dkm204 PMID: 17561500.
14. Lee, M.F.; Peng, C.F.; Lin, Y.H.; Lin, S.R.; Chen, Y.H. Molecular diversity of class 1 integrons in human isolates of *Aeromonas* spp. from southern Taiwan. *Jpn J Infect Dis*. **2008**, *61*, 343-349. PMID: 18806339.
15. Dallenne, C.; Da Costa, A.; Decré, D.; Favier, C.; Arlet, G. Development of a set of multiplex PCR assays for the detection of genes encoding important beta-lactamases in Enterobacteriaceae. *J Antimicrob Chemother*. **2010**, *65*, 490-495. https://doi.org/10.1093/jac/dkp498 PMID: 20071363.
16. Pérez-Pérez, F.J.; Hanson, N.D. Detection of plasmid-mediated AmpC beta-lactamase genes in clinical isolates by using multiplex PCR. *J Clin Microbiol*. **2002**, *40*, 2153-2162. https://doi.org/10.1128/jcm.40.6.2153-2162.2002 PMID: 12037080.
17. Wu, C.J.; Chen, P.L.; Wu, J.J.; Yan, J.J.; Lee, C.C.; Lee, H.C.; Lee, N.Y.;, Chang, C.M.; Lin, Y.T.; Chiu, Y.C.; Ko, W.C. Distribution and phenotypic and genotypic detection of a metallo-β-lactamase, CphA, among bacteraemic *Aeromonas* isolates. *J Med Microbiol*. **2012**, *61*, 712-719. PMID: 22322339.
